# Dexamethasone may affect the occurrence of parenteral nutrition-associated cholestasis in preterm neonates

**DOI:** 10.3389/fped.2022.1023798

**Published:** 2022-12-08

**Authors:** Saizhi Jiang, Qingqing Hu, Jing Zhang

**Affiliations:** Department of Pediatrics, The First Affiliated Hospital of Wenzhou Medical University, Wenzhou, China

**Keywords:** extremely low birthweight, postnatal period, parenteral nutrition associated cholestasis, glucocorticoids, liver disease

## Abstract

**Introduction:**

Glucocorticoids are currently used for the co-therapeutic management of autoimmune hepatitis and some cholestatic diseases. Thus far, we do not know the efficacy of glucocorticoids in the treatment of parenteral nutrition-associated cholestasis. We aimed to analyze whether the administration of late postnatal dexamethasone for treating bronchopulmonary dysplasia influence the occurrence of parenteral nutrition-associated cholestasis in preterm neonates.

**Methods:**

A retrospective study was conducted for 78 preterm neonates without major anomalies (gestational age was <30 weeks, and birthweight was ≤1000 g) hospitalized in a neonatal unit. Total and direct serum bilirubin levels were measured about every two weeks for all neonates. Data including the administration of dexamethasone, intravenous nutrition, and enteral feeding were collected by at least three audits.

**Results:**

A total of 15 preterm neonates were diagnosed with parenteral nutrition-associated cholestasis, and after stopping parenteral nutrition, the direct bilirubin value decreased to the normal level for no longer than 150 days. The prolonged duration of parenteral nutrition was a risk factor, and late postnatal dexamethasone treatment was a protective factor in reducing the incidence of parenteral nutrition-associated cholestasis.

**Conclusion:**

Dexamethasone treatment may reduce the occurrence of parenteral nutrition-associated cholestasis in preterm neonates.

## Introduction

Parenteral nutrition-associated cholestasis (PNAC) is a life-threatening complication in infants and children who must receive long-term parenteral nutrition (PN) due to severe intestinal dysfunction and gastrointestinal feeding intolerance (1), especially in preterm neonates (2). The resolution of cholestasis occurs in some neonates after stopping PN, but some neonates have persistent liver dysfunction and residual fibrosis, leading to liver failure (2, 3). Due to the lack of effective prevention and treatment strategies for PNAC, once PNAC children progress to severe liver failure, if they do not receive organ transplantation, it will lead to high mortality (2, 3). Thus, effective methods to prevent or treat PNAC and reduce liver transplantation still need continuous exploration.

Glucocorticoids are currently used for the co-therapeutic management of autoimmune hepatitis (4) and some cholestatic diseases. Hence, glucocorticoids are recommended for the adjuvant treatment of severe drug-induced liver injury (DILI) with elevated serum total bilirubin level (5) and biliary atresia in neonates after surgery (6). The use of glucocorticoids remains an area of active investigation. Thus far, we have no experience with glucocorticoid use in the treatment of PNAC in neonates, and we do not know the efficacy of glucocorticoids in PNAC treatment. Since dexamethasone (DXM) has been used in the treatment of bronchopulmonary dysplasia (BPD) in preterm neonates, who also need long-term intravenous nutrition support, an analysis of data of these preterm neonates may obtain the effects of glucocorticoid administration on PNAC. As far as we know, there is currently no research in this area. Whether glucocorticoid treatment affects PNAC occurrence is the research content of our team.

## Materials and methods

### Study design and population

This was a retrospective cohort study of patients hospitalized in the First Affiliated Hospital of Wenzhou Medical University between January 2017 to March 2022. The study was restricted to preterm neonates whose gestational age and birthweight were <30 weeks and ≤1000 g, respectively, without major anomalies.

In the absence of other known causes of direct hyperbilirubinemia, PNAC is clinically diagnosed when serum direct bilirubin level is ≥2 mg/dl and >20% of total serum bilirubin in neonates who have received PN for at least 14 days (7). Exclusion criteria included neonates who developed direct hyperbilirubinemia within two weeks of being on PN and those with known causes of direct hyperbilirubinemia, such as (1) TORCH (toxoplasmosis, syphilis, rubella, cytomegalovirus, herpes, human immunodeficiency virus, and parvovirus) infections; (2) chromosomal disorders; (3) metabolic disorders, such as galactosemia and tyrosinemia; and (4) surgical abnormalities of the hepatobiliary system, such as biliary atresia and choledochal cyst (8).

### Enteral feeding and PN administration

The standardized nutritional support management process for preterm neonates was as follows. PN was initiated immediately after birth and consisted of dextrose, protein (6% amino acid injection, China Resources Group, Beijing, China), lipid solution (Lipofundin MCT 20%, B. Braun Melsungen AG, Uppsala, Germany), vitamins, and minerals. Enteral feeding was introduced when the infant was clinically stable, and the enteral nutrition was increased gradually (9).

Intravenous glucose administration began on the first day of life with a dose of 4–6 mg/kg/min and gradually increased to 8–10 mg/kg/min (up to 12 mg/kg/min) according to blood glucose levels (10). The dose of amino acid was increased gradually in increments of 0.5–1.0 g/kg/d on the first day to reach a target dose of 3–4 g/kg/d (11). Intravenous lipid administration was started on day 2 of life at 0.5–1.0 g/kg/d, and the dose was gradually increased to reach a target dose of 3 g/kg/d unless there was hypertriglyceridemia (12). Vitamins and other minerals were provided from the first two days after birth. The intravenous nutrient solution was infused evenly by peripheral or central venous micropump for 24 h. PN was usually stopped once enteral nutrition of 130 ml/kg/d was achieved.

### Evaluation of PNAC

Total and direct serum bilirubin levels were measured every two weeks for all neonates. For neonates who developed direct hyperbilirubinemia (direct serum bilirubin level ≥2 mg/dl and >20% of total serum bilirubin), necessary laboratory investigations, including liver ultrasound, were performed to identify possible causative factors.

Neonates who developed PNAC (defined as serum direct bilirubin level ≥2 mg/dl and >20% of total serum bilirubin after at least 14 days of PN) were classified into the PNAC group, while neonates with a direct bilirubin of <2 mg/dl comprised the control group.

### Late (after seven days) postnatal administration of DXM in preterm neonates

For preterm neonates who still need mechanical ventilation support after two weeks of birth (13), the respiratory status was evaluated (14). After discussion within a neonatal department, the decision of whether to give DXM treatment was made. The initial DXM dose of 0.15 mg/kg/d was injected intravenously for three days, reduced to 0.10 mg/kg/d for three days, then reduced to 0.05 mg/kg/d for two days, and finally reduced to 0.02 mg/kg/d for two days. The whole course of treatment lasted ten days (15).

### Data collection

As we created a database, trained medical record analysts analyzed the following information of preterm neonates from inpatient electronic records and entered data into the database: gender, birthweight, gestational age, information on PN administration and enteral nutrition, DXM treatment, BPD and infection history (16, 17). At least three audits were conducted during the data collection process by 3 of us (JSZ, JZ, and HQQ), and each case data were checked by 3 of us to ensure the accuracy of data.

### Statistical analysis

Proportions were compared by Chi-square, and means were compared by the independent t-test. Binary logistic regression was used directly to analyze the risk factors affecting PNAC. A *P* of <0.05 was considered statistically significant. The sample size of the study was calculated as more than 15 times the number of independent variables participating in logistic regression. All analyses were completed with SPSS 16.0. Figures were made by GraphPad Software (Prism version 5, San Diego, CA, USA).

## Results

**Figure 1:**
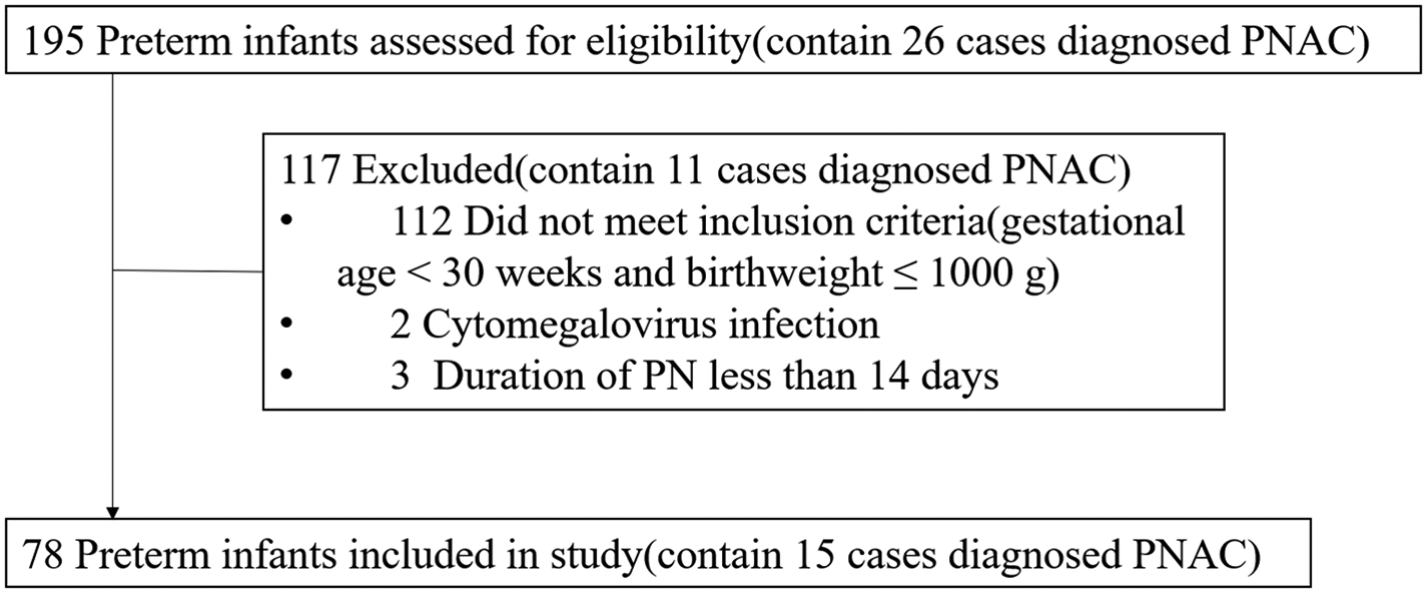
Flow diagram of preterm neonate selection process. PNAC, parenteral nutrition-associated cholestasis; PN, parenteral nutrition.

Between January 2017 and March 2022, according to the inclusion and exclusion criteria, 78 preterm neonates (birthweight ≤1000 g and gestational age <30 weeks, without major anomalies) were finally included in the study (Figure 1). Of the 78 preterm neonates, the percentage of male neonates was less than that of females. Fifteen preterm neonates were diagnosed with PNAC, and there was no significant difference in gender between the PNAC and non-PNAC groups. Birthweight and gestational age between the two groups were similar. In the PNAC group, the average age to start enteral nutrition was higher than that in the non-PNAC group, but there was no statistically significant difference between the two groups, which might be due to the big variation in the PNAC group. The duration of PN in the PNAC group was significantly longer than that in the non-PNAC group. The average dosage of amino acid and fat in the PNAC group was significantly higher than that in the non-PNAC group. However, there was no significant difference in glucose consumption between the two groups. The percentage of postnatal DXM treatment was significantly lower in the PNAC group than in the non-PNAC group: within 15 PNAC neonates, only one neonate received DXM treatment. The proportion of prenatal DXM treatment did not have statistical difference between the two groups. Almost all preterm neonates had a history of infection and BPD; thus, there was no difference regarding infection incidence between the two groups. Additionally, there was no statistical difference regarding the incidence of late-onset sepsis between the two groups. Of the 78 preterm neonates included in the study, only one suffered from neonatal necrotizing enterocolitis (NEC) (the neonate was fed on the fourth day after birth, with PN lasting for 50 days, received DXM treatment, and did not suffer from PNAC) (Table 1).

**Table 1:** Characteristics and potential risk factors in preterm neonates with and without PNAC.

Factors	Total (*n* = 78)	Non-PANC (*n* = 63)	PNAC (*n* = 15)	*P* value
Gender (male/total %)	35.9% (*n* = 28)	39.7% (*n* = 25)	20.0% (*n* = 3)	
Birthweight (g)^c^	885.7 ± 100.6	875.1 ± 102.0	930.7 ± 82.9	
Gestational age (wks)^c^	27.4 ± 1.3	27.3 ± 1.3	27.9 ± 1.3	
Begin of EN (days old)^c^	3.6 ± 2.2	3.5 ± 1.7	4.4 ± 3.4	
Duration of PN (d)^c^	34.9 ± 13.2	33.0 ± 13.0	42.8 ± 11.1	0.009^a^
Average AA dosage (g/kg/d)^c^	2.0 ± 0.4	1.9 ± 0.4	2.2 ± 0.3	0.011^a^
Average Fat dosage (g/kg/d)^c^	1.8 ± 0.4	1.7 ± 0.5	2.0 ± 0.3	0.009^a^
Average Glucose dosage (g/kg/d)^c^	8.0 ± 1.8	7.9 ± 1.9	8.5 ± 1.1	
Postnatal DXM treatment, %	46.2% (*n* = 36)	55.6% (*n* = 35)	6.7% (*n* = 1)	0.001^b^
Prenatal DXM treatment, %	76.9% (*n* = 60)	81.0% (*n* = 51)	60% (*n* = 9)	
BPD history, %	98.7% (*n* = 77)	98.4% (*n* = 62)	100% (*n* = 15)	
Infection history, %	98.7% (*n* = 77)	98.4% (*n* = 62)	100% (*n* = 15)	
Late-onset sepsis, %	69.2% (*n* = 54)	69.8% (*n* = 44)	66.7% (*n* = 10)	
NEC history, %	1.3% (*n* = 1)	1.6% (*n* = 1)	0.0% (*n* = 0)	

^a^ *P* value determined using an independent *t*-test.

^b^ *P* value determined using Chi-square test.

^c^ mean ± standard deviation.

PNAC, parenteral nutrition-associated cholestasis; PN, parenteral nutrition; EN, enteral nutrition; AA, amino acid; DXM, dexamethasone; BPD, bronchopulmonary dysplasia; NEC, neonatal necrotizing enterocolitis.

Based on the results of bivariate analyses in Table 1, the duration of PN, DXM treatment, and average dosage of amino acid and fat were included in the regression analysis (Table 2). Duration of PN and DXM treatment were the only two statistically significant factors. The prolonged duration of PN was a risk factor for PNAC occurrence, and DXM treatment was a protective factor in reducing PNAC incidence. The average dosage of amino acid and fat did not show a statistically significant influence on PNAC incidence in regression analysis.

**Table 2:** Binary logistic regression analysis for the prediction of PNAC.

Variables	beta	Standard error	*P* value	wald	Exp (B)
Duration of PN	0.077	0.031	0.012	6.297	1.080
DXM treatment	−2.739	1.143	0.017	5.739	0.065
Average AA dosage	0.290	1.170	0.804	0.061	1.336
Average Fat dosage	1.628	1.211	0.179	1.806	5.094
Constant	−7.304	2.605	0.005	7.862	0.001

DXM, dexamethasone; PN, parenteral nutrition; AA, amino acid.

Factors with statistical differences in Table 1 and serum direct bilirubin data of 15 PNAC preterm neonates were listed in Table 3 to find out the factors that may affect the severity of PNAC. However, the number of cases was too small for statistical analysis, and the data only provided reference information. Case 12 was not the only case treated with DXM (on the 26th day after birth) but also the case with the lowest serum direct bilirubin value in the PNAC group. The duration of PN in case 12 was 33 days, which was less than the average PN duration in the PNAC group (42.8 d, Table 1), but the beginning of enteral nutrition in case 12 was the latest in the PNAC group. Case 9 had the most severe cholestasis, and the patient started receiving enteral nutrition 8 days after birth and received the highest average fat and glucose dosage, while her PN duration was close to the average (42.8 d, Table 1). Case 1 had the longest duration of PN and the largest average usage of amino acid, but her cholestasis was not serious. It can be inferred from the current data that the severity of cholestasis was affected by multiple factors.

**Table 3:** Description of PNAC cases.

Case No.	DPN (d)	BEN (days old)	DXM (Y/N)	Average dosage of nutrients (g/kg/d)			PNAC (days old)		PDB (μmol/l)
				AA	Fat	Glucose	Begin	End	
1	62	3	N	2.6	2.0	8.0	57	85	47
2	56	2	N	2.0	2.3	6.2	41	98	63
3	53	3	N	2.3	1.9	9.7	27	110	117
4	51	6	N	2.2	1.7	9.8	26	100	107
5	49	4	N	2.1	2.2	7.7	45	134	186
6	45	3	N	2.6	2.2	9.2	29	90	75
7	45	5	N	2.1	1.9	7.7	30	125	158
8	45	4	N	2.1	1.8	6.9	40	85	54
9	44	8	N	2.5	2.5	9.8	27	125	225
10	42	3	N	2.6	2.0	8.9	66	80	43
11	38	3	N	2.2	2.3	8.6	34	91	55
12	33	15	Y	2.2	1.7	9.8	38	99	37
13	29	2	N	1.9	1.9	8.5	25	90	61
14	27	2	N	1.7	1.7	8.5	27	150	213
15	23	3	N	2.5	2.3	8.3	30	100	48

PNAC, parenteral nutrition-associated cholestasis; DPN, duration of parenteral nutrition; BEN, beginning of enteral nutrition; DXM, dexamethasone; AA, amino acid; PDB, peak value of direct bilirubin; Y/N, yes/no.

**Figure 2:**
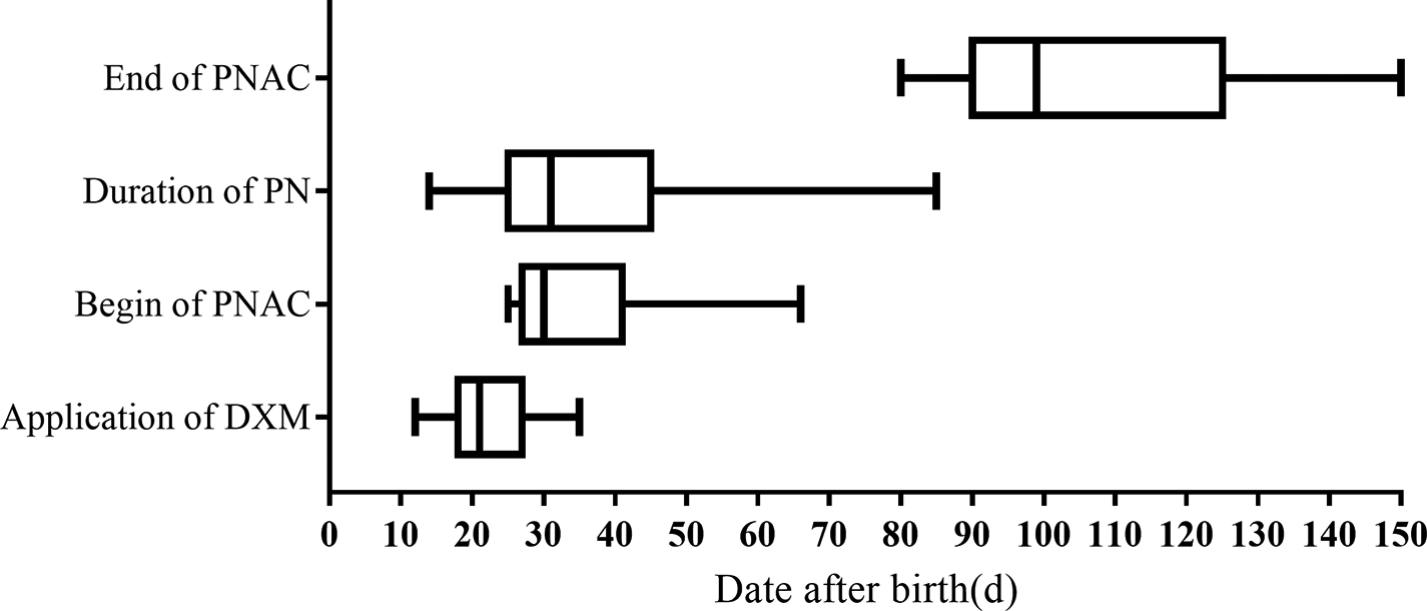
Age distribution of dexamethasone application, duration of parenteral nutrition, and parenteral nutrition-associated cholestasis. DXM, dexamethasone; PNAC, parenteral nutrition-associated cholestasis; PN, parenteral nutrition.

The chronological order of the time to begin DXM treatment (36 cases), duration of PN (78 cases), and development of PNAC (15 cases) are shown in Figure 2. The earliest age at which DXM was administrated was 12 days, the latest was 35 days, and the median age of DXM administration was about 21 days. The median time of PNAC beginning was about 31 days, the earliest age of receiving DXM was 25 days, and the latest age of receiving DXM was 66 days. The application of DXM was earlier than that of PNAC onset. The variation in PN duration was large, with the shortest being 14 days and the longest being 85 days, and the median duration was about 32 days. PNAC was cured after the PN was stopped, with a median age of 100 days (range, 80–150 days).

## Discussion

Mechanisms of PNAC, although studied by many researchers, still remain unclear but are supposedly multifactorial. However, numerous risk factors have been recognized, including preterm birth, short bowel syndrome, lack of enteral feeding, bacterial overgrowth, infection, nutrient deficiency, and nutrient toxicity (18). The incidence of cholestasis in preterm neonates varies greatly with the degree of prematurity, birth weight, and presence of additional risk factors (2). With the advance of enteral feedings and the reduction and discontinuation of PN, the natural course of PNAC in most preterm neonates without intestinal disease is complete biochemical regression (7). The 15 cases diagnosed with PNAC in this study were not complicated by NEC. After stopping PN, the direct bilirubin value decreased to the normal level at the age of 61 days at the earliest and lasted for no longer than 150 days. In this study, preterm neonates with a gestational age of <30 weeks and birthweight of ≤1000 g were selected, and there was no difference in gestational age and birthweight between the PNAC and the non-PANC groups; hence, the effects of gestational age and birthweight were controlled. Although the overall number of female preterm neonates was bigger than that of male preterm neonates, there was no gender difference between the two groups.

Numerous risk factors for PNAC have led to various hypotheses to explain its pathogenesis. It is now believed that biologically active factors absorbed from the injured and hyperpermeable intestine, combined with components of PN solutions (19), synergize to cause hepatic inflammation, cholestasis, and subsequent fibrosis (7). Clinicopathological features of PNAC include cholestasis, variable portal vein inflammation, steatosis, and, ultimately, fibrosis and cirrhosis (20). It has been recognized that in many clinical situations, inflammation either causes or contributes to cholestatic liver disease (21). Due to the strong anti-inflammatory effect of glucocorticoids, these drugs have been used in the treatment of several liver diseases, although their use remains controversial. Glucocorticoids have a potential therapeutic effect on DILI and are recommended for severe DILI patients in cases where the patient's total serum bilirubin levels are exacerbated under regular therapy (5). One study has demonstrated that low-dose DXM can reduce cholestasis-induced liver dysfunction, inflammation, and oxidative stress (22). Adjuvant steroid therapy has become popular for the postoperative management of biliary atresia in infants (6, 23) because steroid therapy improves jaundice clearance. However, it has been questioned that postoperative steroid therapy did not bring certain benefits for patients with biliary atresia in some studies (24). Due to the inconsistency of previous research results, the impact of postoperative steroid treatment on the prognosis of biliary atresia patients is questionable. A clinical case report found that progressive familial intrahepatic cholestasis type 2 patients may benefit from steroid-based therapy (25).Our study found that the early use of low-dose DXM was related to reduced PNAC incidence in preterm neonates. At present, steroid therapy for infantile cholestasis is worthy of further exploration, including the therapeutic effect of steroids on various cholestasis diseases in infancy, as well as the selection of steroid drugs, dosage and duration of use.

Due to the accelerating infant lung maturation and anti-inflammatory effects of glucocorticoids, these medications have been used in the treatment of preterm neonates with chronic pulmonary dysplasia for decades (13), providing a very obvious life-saving effect. Our data analyzed the effect of DXM treatment for BPD on the incidence of PNAC in preterm neonates. DXM treatment was administered before PNAC developed, i.e., in the late stage after birth. The neonatal liver is relatively immature, especially in preterm neonates, and during the postnatal period, the liver undergoes several changes in its functional capacity. Preterm neonates are susceptible to the effects of immature liver function, placing them at risk of hyperbilirubinemia, cholestasis, and impaired drug metabolism (26). This might be the reason why preterm neonates are more prone to PNAC than term neonates.

In addition to the strong anti-inflammatory effect, glucocorticoids are also known to stimulate maturation in many differentiating tissues, including the lung, liver, and heart (27). Studies in animals have shown that prenatal glucocorticoid administration leads to hepatocyte maturation in preterm fetuses (22, 23). Studies conducted *in vivo* and *in vitro* have indicated that glucocorticoids, e.g., dexamethasone, affect the expression of several genes involved in the synthesis and enterohepatic cycling of bile acids, that might lead to increased bile acid synthesis and their altered enterohepatic cycling (28). We did not research the mechanism of DXM affecting the occurrence of PNAC in this study. Moreover, the study was a single-center retrospective study, and the number of cases was small, reducing the quality of the research results. There were not enough NEC cases to analyze the impact of DXM on PNAC occurrence, which was also one of the defects of this study. We speculate that early DXM administration might reduce PNAC occurrence in preterm neonates by promoting the maturation of the liver and coordinating anti-inflammatory effects.

In conclusion, our data indicated that DXM administrated in the late postnatal period of preterm neonates for the purpose of treating BPD might reduce PNAC occurrence in the later period. Based on our conclusions, we recommend that for preterm neonates with persistent ventilator dependence, DXM treatment could be introduced more actively if there is a high risk of PNAC. However, our findings should be considered preliminary, and further research is required to assess long-term and clinical outcomes. If our findings are verified in the future, the application of DXM will improve the survival rate of very low birth weight preterm neonates and reduce the incidence of complications.

## Data Availability

The raw data supporting the conclusions of this article will be made available by the authors, without undue reservation.
